# Contributions of forensic anthropology to positive scientific identification: a critical Review

**DOI:** 10.1080/20961790.2018.1523704

**Published:** 2018-10-08

**Authors:** Douglas H. Ubelaker, Austin Shamlou, Amanda Kunkle

**Affiliations:** Department of Anthropology, Smithsonian Institution, Washington, DC, USA

**Keywords:** Forensic sciences, forensic anthropology, positive scientific identification, medicolegal death investigation, craniofacial superimposition, facial approximation, team approach

## Abstract

This review covers previous and current literature on the impact of forensic anthropologists on the positive scientific identification of human remains and aims to provide an understanding of what information a forensic anthropologist can contribute to an investigation. Forensic anthropologists looking to identify human remains study traits of the skeleton and any orthopedic devices present. In order to obtain a positive scientific identification, evidence that is both sufficiently unique to the individual and comparable to available antemortem data from that individual must be found. The increased availability of radiographs, scans and implants in recent decades has facilitated the identification process. When these records are unavailable, other techniques, such as craniofacial superimposition and facial approximation, can be employed. While these methods may assist the identification process, they are most useful for exclusion of certain individuals and gathering leads from the public. Forensic anthropologists have heavily relied on the skull and its complexities for identification – typically focusing on the frontal sinus and other unique traits. Post-cranial remains can provide important information about bone density, possible disease and other characteristics that may also be utilized. Techniques used to positively identify individuals are not limited to medicolegal death investigations, and have been useful in other legal contexts. In the future, a team approach, utilizing all the information gathered by multiple forensic scientists – including forensic anthropologists – will most likely become more common.

## Introduction

Positive scientific identification of human remains recovered in a medicolegal context represents a central goal of forensic anthropology analysis. Many facets of anthropological activity, including search and recovery, determining species, estimation of sex, age at death, stature, time since death, and ancestry and detection of unique anatomical features produce information used to narrow the search of missing persons. Ultimately, forensic anthropologists contribute to positive scientific identification either directly, or through the wealth of supplemental information provided. Direct contributions involve assessment of a variety of anatomical features and comparison with antemortem information, usually revealed through radiography and related imagery.

Types of identification include tentative, circumstantial, presumptive and positive types [1]. The first three types listed indicate that the actual identification cannot be excluded and thus the remains, or other evidence examined, might represent a particular individual. Research and casework has demonstrated that facial recognition is generally unreliable in identification, especially with advanced decomposition [2].

Positive identification represents a much higher level of probability and involves a two-step process. First, anatomical features must be discovered that are shared between the examined evidence and the known antemortem information relating to a particular individual. Second, the analyst must determine that the features being compared are sufficiently unique to enable the identification. In addition, any differences must be noted and explained in a satisfactory manner. When errors are made in identification they usually fall into two categories: (1) differences are considered as evidence for exclusion that actually represent other factors, and (2) shared features are presented in support of identification with insufficient consideration of their uniqueness. Great caution is needed in interpretation since misidentification can lead to tragic consequences.

Contributions of forensic anthropologists to identification are especially needed and valuable in the analysis of extensively decomposed and/or skeletonized human remains. Experimental research reported by Sauerwein et al. [3] indicates that the process of decomposition can rapidly destroy many indicators commonly used in identification, although the rate of destruction depends on many variables. In their research, fingerprints survived 4 days postmortem with warm temperatures but more than 50 days with cold temperatures. Postmortem iris recognition ranged from 2 to 34 days, depending on the variables involved. Of course, this research is location specific and rates may vary in other regions.

Proper recovery, documentation and assessment of the biological profile of human remains are essential elements leading to positive identification [4]. Details of this methodology are beyond the scope of this article; however, shortcomings of any aspect of these procedures can prevent identification and/or derail investigation. To focus on the proper set of missing persons for possible identification, investigators must have meaningful information on the age at death, sex, ancestry, stature and time since death [5]. All of the evidence must be recovered with detailed documentation. Both the recovery and analysis must be conducted in a manner to meet the demands of the legal process [6, 7].

The unique features needed for positive identification can be provided by surgical procedures, especially those generating devices that remain in the skeletal tissue [8]. For example, Hogge et al. [9] were able to positively identify remains through their detection of post-surgical defects relating to a unilateral lambdoid synostectomy. The remains were identified as an individual who had undergone neurosurgery for this rare congenital anomaly.

Many orthopedic devices recovered with human remains may present information revealing the manufacturer [10]. Some devices, following current law, may include numerical information that can be traced to a particular surgical office or even the individual patient.

Forensic anthropologists have found these inorganic materials fundamental in their cases. For example, Bennett and Benedix [11] report positive identification of burned remains recovered from an automobile through detection of an internal fixation device. Radiographic examination of recovered remains revealed a complex of wires that were determined to represent an osteostimulator. No serial number was noted, but the metal materials related to a documented osteostimulator surgically employed to stimulate bone production in treatment of a back injury of a patient.

When lacking surgical modifications, anthropologists must look for organic anomalies or unique traits in skeletal remains. The classic text of Stewart [12] discussed positive identification in a chapter entitled “Traits Peculiar to the Individual.” Stewart noted the value of unique, highly variable anatomical features in identification. He also recognized the importance of careful cleaning of remains to facilitate observation of such features. Hogge et al. [13] later called attention to the value of experience in recognition and interpretation of anatomical features used in identification.

Dental features frequently provide information needed for identification [14]. Forensic odontologists are uniquely qualified to interpret dental restorations and other features related to the practice of dentistry. However, anthropologists share with odontologists an interest and expertise in aspects of dental morphology that can provide evidence for positive scientific identification. Useful features include the number of teeth present, antemortem loss of teeth, patterns of displaced teeth and patterns of unusual rotation [15].

Comparative antemortem information is usually available through radiographs and related imagery. Murphy and Spruill [16] report that in a 15-month period from April 1978 to July 1979 in the St. Louis area of the United States, 60% of scientific identifications resulted from radiographic assessment. Anatomical variants, disease modification and postsurgical features provided most of the unique data utilized in positive scientific identification. As noted by Fitzpatrick and Macaluso [17], techniques of positioning, magnification, beam centering, angulation and bone orientation must be employed properly to facilitate comparison.

## Craniofacial superimposition

Craniofacial superimposition compares features of a recovered skull thought to be of medico-legal interest with antemortem photographs of a missing person who might be represented by the remains. This technique may be employed when positive identification has not been accomplished through molecular analysis, dental reconstruction comparison or anthropological radiographic assessment [18]. Usually, the method is utilized when complete skulls or crania are available for comparison [19], but attempts have been made using even fragmentary evidence [20]. Once clear images are found that can be used to compare the recovered crania, forensic anthropologists must take the time to orient the skull, often using Q-tips as place markers, in order to be able to lay the images properly over each other [21]. The techniques of comparison have become more complex and sophisticated [18, 19] but primarily allow exclusion rather than positive scientific identification. Images are often pulled from police records, surveillance or directly from relatives of the possible individual. The quality of this image typically corresponds to the accuracy of the exclusion process [21].

Dorion [22] notes that photographic superimposition can lead to misidentification if not properly employed. He cautions that the technique should not be used as the only means of identification. Research reported by Austin-Smith and Maples [23] supports this expression of concern. They compared frontal and lateral views of three skulls with photographs of 98 different persons. They reported a positive comparison with 9.6% of the lateral views and 8.5% of the frontal views. However, the percentage of consistency was reduced to 0.6% when both frontal and lateral views were utilized.

## Facial approximation

Facial approximation represents the attempt to produce a facial likeness of an individual from a skull. While the method cannot be used for positive identification directly, the image produced can be used to communicate with the public in an effort to gather information on missing persons who might be represented by the recovered remains. Major advances in methodology include new population data of soft tissue depth, new guidelines of assessing facial features and innovative computerized approaches [24]. Although multiple studies of soft tissue depth have been published, Stephan and Simpson [25] note that the data indicate no clear secular trends and the values have wide variation. They suggest that existing data be pooled for use with adults. Stephan and Simpson [26] also found similar results with subadult data and recommended categorizing the data into two age groups (0–11 years and 12–18 years).

Although facial approximations have been reported to be useful in gathering information relative to identification, Stephan and Cicolini [27] reported concern about the associated resemblance ratings. Stephan and Henneberg [28] published an experimental approach to judge the recognition value and questioned the value for identification.

Techniques of facial approximation are improving with enhanced information regarding the relationship of facial hard and soft tissues and more sophisticated computer technology. Despite these advancements, facial approximation does not represent a method of positive scientific identification. However, the generated image may prove useful to assist public communication that the remains of someone with particular visual and demographic characteristics have been recovered.

## Unique cranial evidence

While other methods have led to tentative identifications, distinctive features present on the skeleton allow for a more certain classification. The skull frequently has provided the unique information needed for positive scientific identification in anthropological analysis for two primary reasons: (1) historically, considerable research has focused on the skull revealing great variation of many anatomical features, and (2) antemortem radiographs and related imagery frequently are available for the head and may include multiple views. As noted by Smith et al. [29], skull images can present numerous unique features useful for identification. In their case report, Smith et al. [29] indicated that positive scientific identification was enabled by computerized tomographic (CT) examination of the frontal sinus, sphenoid sinus, ethmoidal mastoid air cells, the sagittal suture and aspects of the internal occipital protuberance. Culbert and Law [30] provide a very early reference to their use of radiographic examination of nasal accessory sinuses and the mastoid processes for identification of a former patient who died in India. Rhine and Sperry [31] provide an additional example of identification using the frontal sinuses and endocranial arterial patterns. Rogers and Allard [32] also employed cranial suture patterns (location, length and slope of sutural lines) and argued their approach to these features met legal requirements in the United States and Canada at that time.

## Frontal sinus variation

Although many features of the human skull display extensive variation and thus are useful for individual identification, many investigators have focused on the frontal sinus. This sinus located superior to nasion in the area of the supraorbital ridges displays remarkable variation ranging from minimal presence to large labaryinthion formations. Apparently reflecting environmental and developmental influences, even identical twins display morphological differences in frontal sinus expression [33].

As early as 1921, Schϋller [34] called attention to the value of the frontal sinus for positive scientific identification. Later, Asherson [35] developed a system of using outlines of the sinus expression for comparative purposes. In 1984, Ubelaker [33] described how frontal sinus comparison, coupled with morphology of the sella turcica and other cranial features, was utilized for positive scientific identification in a murder trial. Ubelaker [33] also used radiographs of cranial collections at the Smithsonian Institution to demonstrate population variation of the frontal sinus. Angyal and Dérzy [36] presented cases from Hungary showing how radiography of the frontal sinus, along with features of the pelvis, humerus and lumbar vertebrae, allowed positive scientific identification.

Although most early comparative studies of frontal sinus morphology utilized in medicolegal applications featured pattern recognition, metric and more sophisticated statistical treatments have been introduced as well. Kirk et al. [37] introduced a metric approach that documented the vertical and horizontal dimensions of the sinus expression. They declared a match if the comparative measurements were within 5 mm of each other. In their retrospective study of 39 cases from the Ontario Chief Coroner’s Office, Kirk et al. [37] reported using both pattern recognition and their metric analysis for positive scientific identification. In addition, they reported that adult age, sex and cause of death had no effect on the likelihood of identification using this feature.

Noting the growing demands from the legal arena for increased quantification and probability assessment of features used in identification, Christensen [38] applied Elliptic Fourier analysis in assessing the individualization of the frontal sinus. As suggested in the literature published earlier, this application indicated that assessment of frontal sinus morphology represented a reliable approach to human positive scientific identification.

## Post-cranial remains

Skeletal remains from the post-cranial skeleton also present abundant anatomical features useful for identification if corresponding antemortem radiographs can be located. Post-cranial bones may be less affected by animal scavenging and other postmortem factors [39]. General trabecular bone patterns [40], as well as general bony contours, anomalies and radiodensities [41] can provide unique features useful for identification. Post-cranial approaches to identification have focused on the clavicle [42–46], general chest area [47–49], hand and wrist [50], patella [51] and foot deformity [52]. Unusual medical conditions are important since they often can be linked to radiographs showing skeletal anatomical details.

## Applications to the living

Although contributions of forensic anthropologists to positive scientific identification usually involve recovered skeletal remains, similar techniques can be applied to medicolegal issues involving the living. Fenger et al. [53] report how radiographic evaluations of skeletal details were used to address cases of workers’ compensation fraud. Individuals in Colorado with pre-existing medical conditions were feigning injuries while at work and claiming workers’ compensation. Using different identities, they were making multiple claims for the same apparent medical condition. Comparative examination of radiographs revealed that multiple claims supposedly of different persons actually related to one person.

## Team approach

Although anthropologists frequently apply their skills to individual skeletal features, ultimately identification represents a team effort [54]. Apart from investigative efforts, reports and analyses by forensic anthropologists join those generated from analysis of DNA, fingerprints, dental restorations and other data [55]. Ideally, identification should represent a holistic, comprehensive process that builds on the biological profile and circumstantial evidence.

## Future advances

Critical evaluation of past progress reveals trends likely to produce future advances. Technological advances clearly represent key potential for enhanced capability in positive scientific identification. The images generated by computerized tomography (CT) reveal much more skeletal detail than those previously available from conventional radiography. The rapidly advancing technology available for imagery clearly will contribute to major advances.

Recent years have witnessed increased scrutiny of the forensic sciences in the legal arena. Constructive criticism has stimulated research focus on probability assessment, cognitive bias, error analysis and the general scientific foundation of forensic applications. Future analyses of features contributing to positive scientific identification must relate accurately the probabilities involved. Those involved in the identification process must guard against cognitive bias that might impact assessment. Research must attempt to define the uniqueness of features commonly involved in skeletal identifications. Concepts of “match” and “consistency” likely will be replaced with more precise statements of probability and associated error. More sophisticated statistical analyses predictably will become apparent in the research designs targeting methods of identification.

The team approach discussed above likely will become more commonplace in the identification procedure. Individual techniques and statistical analysis present individual probabilities of identification. The team approach offers the potential for combined probabilities that should enhance the identification effort.

Identification benefits from the training and experience of anthropologists conducting the analysis. Internationally, the best and brightest students are becoming increasingly attracted to the field of forensic anthropology. This surge of academic interest and dedication bodes well for the future of forensic anthropology and for methodology of positive scientific identification.
